# The distribution of MEFV mutations in Turkish FMF patients: multicenter study representing results of Anatolia

**DOI:** 10.3906/sag-1809-100

**Published:** 2019-04-18

**Authors:** N. Şule YAŞAR BİLGE, İsmail SARI, Dilek SOLMAZ, Soner ŞENEL, Hakan EMMUNGİL, Levent KILIÇ, Sibel YILMAZ ÖNER, Fatih YILDIZ, Sedat YILMAZ, Duygu ERSÖZLU BOZKIRLI, Müge AYDIN TUFAN, Sema YILMAZ, Veli YAZISIZ, Yavuz PEHLİVAN, Cemal BES, Gözde YILDIRIM ÇETİN, Şükran ERTEN, Emel GÖNÜLLÜ, Fezan ŞAHİN, Servet AKAR, Kenan AKSU, Umut KALYONCU, Haner DİRESKENELİ, Eren ERKEN, Bünyamin KISACIK, Mehmet SAYARLIOGLU, Muhammed ÇINAR, Timuçin KAŞİFOĞLU

**Affiliations:** 1 Division of Rheumatology, Department of Internal Medicine, Eskişehir Osmangazi University, Eskişehir Turkey; 2 Division of Rheumatology, Department of Internal Medicine, Dokuz Eylül University, İzmir Turkey; 3 Division of Rheumatology, Department of Internal Medicine, Erciyes University, Kayseri Turkey; 4 Division of Rheumatology, Department of Internal Medicine, Ege University, İzmir Turkey; 5 Division of Rheumatology, Department of Internal Medicine, Hacettepe University, Ankara Turkey; 6 Division of Rheumatology, Department of Internal Medicine, Marmara University, İstanbul Turkey; 7 Division of Rheumatology, Department of Internal Medicine, Çukurova University, Adana Turkey; 8 Division of Rheumatology, Department of Internal Medicine, University of Health Sciences,Gülhane Faculty of Medicine, Ankara Turkey; 9 Division of Rheumatology, Department of Internal Medicine, Adana Numune Education and Research Hospital, Adana Turkey; 10 Division of Rheumatology, Department of Internal Medicine, Selçuk University, Konya Turkey; 11 Division of Rheumatology, Department of Internal Medicine, Şişli Etfal Education and Research Hospital, İstanbul Turkey; 12 Division of Rheumatology, Department of Internal Medicine, Gaziantep University, Gaziantep Turkey; 13 Division of Rheumatology, Department of Internal Medicine, Abant İzzet Baysal University, Bolu Turkey; 14 Division of Rheumatology, Department of Internal Medicine, Kahramanmaraş Sütçü İmam University, Kahramanmaraş Turkey; 15 Division of Rheumatology, Department of Internal Medicine, Yıldırım Beyazıt University, Ankara Turkey; 16 Department of Biostatistics, Eskişehir Osmangazi University, Eskişehir Turkey; 17 Division of Rheumatology, Department of Internal Medicine, Medical Park, Gaziantep Turkey

**Keywords:** Familial Mediterranean fever, Mediterranean fever gene mutations, M694V, Turkey

## Abstract

**Background/aim:**

The distribution of Mediterranean fever (MEFV) gene mutations in Turkish familial Mediterranean fever (FMF) patients varies according to geographic area of Turkey. There is a need for highly representative data for Turkish FMF patients. The aim of our study was to investigate the distribution of the common MEFV mutations in Turkish FMF patients in a nationwide, multicenter study.

**Materials and methods:**

Data of the 2246 FMF patients, from 15 adult rheumatology clinics located in different parts of the country, were evaluated retrospectively. The following mutations have been tested in all patients: M694V, M680I, M694I, V726A, and E148Q.

**Results:**

There were 1719 FMF patients with available genetic testing. According to the genotyping, homozygous M694V, present in 413 patients (24%), was the most common mutation . One hundred and fifty-four (9%) of patients had no detectable mutations. Allele frequencies of common mutations were: M694V (n = 1529, 44.5%), M680I (n = 423, 12.3%), V726A (n = 315, 9.2%), E148Q (n = 214, 1%), and M694I (n = 12, <1%).

**Conclusion:**

In this large-scale multicenter study, we provided information about the frequencies of common MEFV gene mutations obtained from adult Turkish FMF patients. Nearly half of the patients were carrying at least one M694V mutations in their alleles.

## 1. Introduction

Familial Mediterranean fever (FMF) is a hereditary autoinflammatory disease with self-limiting attacks characterized by serositis and fever (1). The disease primarily occurs in individuals of Mediterranean ancestry, and particularly among certain ethnic groups such as Jews, Turks, Armenians, and Arabs (2). Turks are considered to have the highest prevalence with an estimated rate of 1:150 to 1:1000 (1,3). FMF is caused by the mutations in the MEFV (Mediterranean fever) gene and inherited in an autosomal recessive manner. However, nearly 30% of documented FMF patients exhibit non-Mendelian genetic transmission, carrying only one mutation, and up to 20% of patients do not have detectable mutations as per current technology (4). So far, more than 300 sequence variations have been identified in the MEFV gene, mostly due to single nucleotide substitutions (5). Five founder mutations, located on exon 10 (M694V, V726A, M694I, and M680I) and exon 2 (E148Q), account for nearly 80% of patients with typical cases from these ethnic groups (1). A previous study of 1090 Turkish FMF patients reported in 2005 showed that M694V was the most frequently observed mutation, followed by M680I and V726A (3). 

MEFV mutations data obtained 12 years ago may not still be accurate because of the limited number of known MEFV mutations at that time. Therefore, there is a need to update MEFV mutation distribution in Turkish FMF patients. Herein, we aimed to investigate the frequencies of mutations in adult Turkish FMF patients in a multi-center study.

## 2. Materials and methods

In this study, we used the data of an FMF registry that includes patients from 15 adult rheumatology clinics located in different regions of Turkey. The first evaluation results generated from the database of FMF patients were published in 2014 (6) and evaluated the relationship between FMF and amyloidosis. The FMF patients in this registry were diagnosed according to the Tel-Hashomer or Sheba Medical Center criteria (7,8) (Because of the retrospective design of the study we were not able to choose one criteria set). There were 2246 patients in the registry. The age range was 34.5 ± 11.9 years, and 46.7% of the group was male. For the current study we included patients who had available genetic testing for MEFV. There were 1719 patients who had available MEFV genotype and demographic information. MEFV tests were performed at the laboratories where participating clinics were located. Each center included in this study has an established technique for determining MEFV. In most centers, genetic testing was mainly done by PCR-RFLP or the reverse hybridization assay (FMF StripAssay). We tested all patients for the founder mutations M694V, M680I, M694I, V726A, and E148Q. For the calculation of allele frequencies we used the formula n/2N, which shows the number of mutations (n) in the MEFV genes of the N screened subjects (9). This study was approved by the local ethical committee and each patient provided written consent before registering in the database.

### 2.1. Statistical analysis

Continuous variables were presented as mean ± SD, and categorical variables were presented as frequency (n) and percent. The chi-square statistic was used for testing relationships between categorical variables. IBM SPSS Statistics 21 was used for descriptive statistics.

## 3. Results

A total of 2246 FMF patients were evaluated. 1719 had available MEFV mutation analysis. Of the latter group, 809 (47%) were male and 910 (53%) were female. The mean age and mean age at symptom onset was 33.4 (±12) and 16.5 (±10.2) years respectively. The delay in diagnosis was 10.2 (±10.7) years (Table 1). 

**Table 1:** Demographic characteristics of FMF patients.

FMF patients (n = 1719)	Mean ± SD
Age, yrs	33.4 ± 12
Male/Female, (%)	809/910, (47/53)
Age at symptom onset, yrs	16.5 ± 10.2
Age at diagnosis, yrs	26.6 ± 12.1
Delay in diagnosis, yrs	10.2 ± 10.7

According to the genotyping, homozygous M694V, present in 413 patients (24%), was the most common mutation. Other common mutations were M694V heterozygous (n: 305, 17.7%), M694V/M680I (n: 144, 8.4%), M694V/V726A (n: 126, 7.3%), M694V/E148Q (n: 91, 5.3%), V726A/M680I (n: 73, 4.3%), E148Q heterozygous (n:72, 4.1%), M680I heterozygous (n:64, 3.7%), M680I homozygous, (n:52, 3%) and heterozygous V726A (n:50, 2.9%). Less common mutations (<1%) were compound heterozygous V726A/E148Q (n:11), M680I/R761H (n:15), E148Q/P369S (n:9), M694V/K695R (n:1), T267I/R314H/H390D (n:1), V726A/F479L (n:8), V726A/P369S (n:1), M694V/P369S (n:2), M694V/V726A/P369S (n:2), M694V/M694I (n:2), E148Q/M694V/P369S (n:3), M694I/M680I (n:1), M694I/M680 (n:1), E148Q/R761H (n:2), M694I/E148Q (n:3), M694V/M680I/E148Q (n:2); homozygous R761H (n:6), M694I (n:3), E148Q (n:1), F479L (n:1), and heterozygous R761H (n:3), K695R (n:3), F479L (n:3), and P369S (n:6). The total frequency of these rare mutations was 11%. One hundred and fifty-four (9%) patients had no detectable mutations, which was classified as a “wild-type” genotype. The list of the mutations is summarized in Figure.

**Figure:**
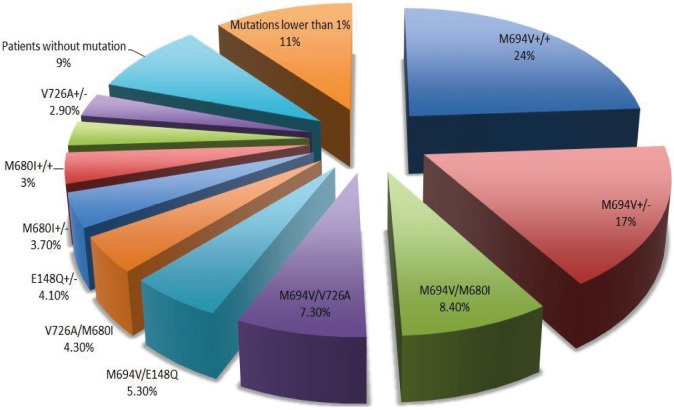
Frequency of MEFV mutations.

 Our testing for founder mutations showed M694V to be the most common mutation (n = 1529, 44.5%), followed by M680I (n = 423, 12.3%), V726A (n = 315, 9.2%), E148Q (n=214, 1%), and M694I (n = 12, <1%)

We then performed a subgroup analysis for the association of clinical and demographical features with common alleles (M694V, M680I, V726A, and M694I; n = 2279), E148Q (n = 214) and wild-type allele (n = 785). According to that analysis fever, arthritis, and amyloidosis were more prevalent in the pathogenic mutations group than the E148Q and wild-type subsets (Table 2). Significantly lower frequencies of elevated liver enzymes (ELE), myalgia, and vasculitis were observed in the E148Q group than the others (Table 2). 

**Table 2:** Association of mutations with clinical features.

	Presence of pathogenic allele (N = 2279)	E148Q allele(N = 214)	Wild-type allele (N = 785)	P
Sex, Males, %	49.2**	46.9	41.4	0.001
Fever, %	92.4*,**	88.2	87.9	<0.0001
Peritonitis, %	94.8	94.4	94.1	0.78
Pleuritis, %	50.4	47.6	46.8	0.08
Arthritis, %	46.4*, **	29.7	36.6	<0.0001
ELE, %	27.5*, **	12.3	20***	<0.0001
Myalgia, %	14.4**	12.7	19***	0.008
Vasculitis, %	6.9*	3.3	9.2***	0.01
Amyloidosis, %	11.4*, **	3.8	2.7	<0.0001

Following indicates significant differences between * common pathogenic allele vs. E148Q, ** common Pathogenic allele vs. wild-type allele and *** E148Q allele vs. wild-type allele. Note that mutations that were not assessed routinely by all study centers (n = 160 allele) not included in the analysis.

## 4. Discussion

FMF is the most common autoinflammatory disease and Turkey has one of the highest incidence and prevalence ratios in the world. The frequency of FMF is reported to be nearly 1% in some geographic regions, particularly central Anatolia, and the overall prevalence is around 0.1% (10). The estimated number of patients in Turkey is approximately 70,000–100,000. The disease is clinically characterized by self-limiting inflammatory attacks. It may also be related to severe complications such as amyloidosis. 

In a previous study using the same registry, we reported the prevalence and risk factors of amyloidosis in Turkish FMF patients. Nearly 10% of the group had amyloidosis. Homozygosity of M694V was the most important genotype associated with this condition (6). In the current study, we investigated the frequency of MEFV mutations in 1719 FMF patients. To our knowledge, this sample size is larger than any studies previously conducted and is meant to reflect all of Turkey. According to the genotype, homozygous M694V (24%) was the most common mutation, followed by heterozygous M694V, compound heterozygosity of M694V/M680I, M694V/V726A, M694V/E148Q, and V726A/M680I; heterozygous E148Q; heterozygous M680I; and homozygous M680I. M694V was the most common allelic mutation (44.5%), followed by M680I (12.3%), V726A (9.2%), E148Q (1%), and M694I (<1%). 

M694V allele in FMF is reported in a range of 20%–65% in different ethnic populations (11). There are many reports with conflicting results about the frequency of MEFV mutations from different geographic regions of Turkey (Table 3). M694V mutation was the most common mutation in studies from central Anatolia (12–15), southeastern Anatolia (16), Aegean regions (17,18), northeastern Anatolia (19), and eastern Anatolia (20,21). A study by Akar et al. examined 230 patients and found the homozygous M694V genotype to be the most common (13). A large-scale study by a Turkish FMF study group, used genetic analysis of 1090 patients and found M694V [51.4% (1121/2180)] to be the most common genetic mutation (3). Another study by Barut et al. evaluated 708 children with FMF and found homozygous M694V (21.8%) to be the most common mutation, followed by heterozygous M694V (%19.2) (22). Studies that included screening for R202Q polymorphism found a higher frequency of this variant than the M694V pathogenic variant; however, it is now accepted that the common R202Q variant is not associated with FMF morbidity (23–25). There are also differences in the freuqency of other common genotypes between the regions. E148Q was the most common mutation in a study from the southeastern region by Ece et al. (26), whereas E148Q was the second most common mutation in the eastern region (16,17,20), northeastern region (19), and central Anatolia (12). The frequency of common FMF-causing alleles in different regions of Turkey is summarized in Table 3.

**Table 3:** Frequency of common FMF-causing alleles in different regions of Turkey.

Study region	Number of patients (pts)	M694V% (n)	V726A% (n)	M680I% (n)	E148Q% (n)	None% (n)	Methods	Reference
Eastern Anatolia	453	36.5 (215)	14.09 (83)	3.9 (23)	32.77 (193)	*	n/N	Coskun et al. (2015)(20)
Eastern Anatolia	415	21.6 (180)	9.7 (81)	9.5 (79)	19.1 (159)	30.7 (255)	n/N	Etem et al. (2010)(21)
Southeastern	147	26 (50)	13 (20)	6.3 (25)	30.7 (59)	*	n/2N (102 null allels were excluded)	Ece et al. (2014) (26)
Southeastern	104	18.3 (19)	8.6 (9)	6.7 (7)	30.8 (32)	14.4 (15)	n/N	Evliyaoglu et al. (2009)(30)
North East (Black Sea)	1620	42.8 (-)	16.3 (-)	14.1 (-)	14.7 (-)	54.9 (889)	n/N	Dogan et al. (2015) (19)
Central Anatolia	2067	14.68 (607)	4.78 (197)	7.62 (385)	5.15 (228)	49.5 (1023)	n/2N	Dundar et al. (2011) (12)
Central Anatolia (Ankara)	230	43 (-)	*	12 (-)	*	15.7 (36)	n/N	Akar et al. (2000)(13)
Central Anatolia	330	50 (330)	9.7 (64)	14.1 (93)	1.36 (9)	20.15 (133)	n/N	Demirkaya et al. (2008)(15)
Central Anatolia	802	14.2 (228)	5 (87)	4 (62)	4 (71)	51.9 (416)	n/2N	Ceylan et al. (2012)(14)
Western Anatolia	383	41.15 (93)	7.08 (16)	12.3 (28)	20.3 (46)	54.3 (208)	n/2N (among 226 alleles)	Coskun et al. (2015)(18)
Aegean Region	1201	47.6 (375)	12.95 (102)	11.94 (94)	16.75 (132)	54.5 (654)	n/2N	Akın et al. (2010) (17)
Mediterranean	1000	7.95 (159)	1.85 (37)	2.4 (48)	8.85 (177)	38.2 (382)	n/2N	Gunesacar et al. (2014) (25)
Overall Turkey	1090	51.4 (1121)	8.1 (188)	14.4 (313)	*	*	n/2N	Tunca et al.(2005) (3)

*frequencies not reported

Delay in FMF diagnosis can significantly increase morbidity and may contribute to an increase in both mortality and healthcare costs. The main reasons for the diagnostic delay can be grouped into physician-related (e.g., lack of knowledge), patient-related (e.g., denial or misinterpretation of symptoms), and disease-related (e.g., atypical clinical findings) factors (27). In our study, the mean delay of diagnosis for our patients was 10.1 ± 10.6 years, which indicates a need to improve early recognition of FMF in the primary care setting.

 Although FMF is typically transmitted via autosomal recessive inheritance, a considerable number of patients may have 1 (up to 30%) or no identifiable mutations (up to 20%) in their MEFV genes (4, 13, 28, 29). In our study, 38% of patients did not show the typical autosomal recessive pattern. Nine percent had no detectable mutation, and 29% had mutation in only one allele. This may be explained by the fact that 1) most of the genetic testing was designed to screen for commonly observed mutations, so rare mutations may not be among those screened for and thus not be present in the results or 2) current diagnostic methods may be insufficient for the detection of all possible mutations. 

Differences between geographic regions may be caused by the wide genetic diversity in our country due to ongoing interactions between different ethnic and cultural groups through history. The frequency of MEFV in Turkish FMF patients is similar to populations in Mediterranean and Middle Eastern countries. Our results showing M694V as the most common mutation among non-Ashkenazi Jews, Arabs, Armenians, and Turks were also reported in a study by Touitou et al. (11). Some relatively rare mutations are more common in certain populations (e.g., V726A is the second most common mutation in Arabs and Ashkenazi Jews) (11). The increased frequency of genetic diseases like FMF, and similar genetic mutations in populations living in the same geographic area for thousands of years, suggests the possibility of genetic interactions. 

In conclusion, in this large FMF patient cohort, we found the prevalence and clinical significance of common MEFV variants. Additionally, we replicated the previous studies showing M694V as the most common pathogenic mutation in Turkish FMF patients.
